# Assessment of Pb, Cd, As and Hg concentration in edible parts of broiler in major metropolitan cities of Tamil Nadu, India

**DOI:** 10.1016/j.toxrep.2021.03.017

**Published:** 2021-03-23

**Authors:** Mathaiyan M, Natarajan A, Xavier Rajarathinam, Rajeshkumar S

**Affiliations:** a Department of Chemistry, Research and Development Centre, Bharathiar University, Coimbatore, India; b Animal Feed Analytical and Quality Assurance Laboratory, Veterinary College and Research Institute, Tamil Nadu Veterinary and Animal Sciences University, Namakkal, India; c Department of Chemistry, Sacred Heart College Tirupattur, Tamil Nadu, India; d Department of Pharmacology, Saveetha Dental College and Hospitals, SIMATS, Chennai, 600077, Tamil Nadu, India

**Keywords:** Heavy metals, Broiler chicken, ICP-MSMS, Residue analysisis, Metropolitans and MRL

## Abstract

The risk of heavy metals contamination in meat and meat products is of great concern for both food safety and from human health point of view. The present study was carried out to assess the heavy metal residues such as Pb, Cd, As and Hg in broiler chicken meat, neck and edible organs (liver and kidney). In the present study, 150 samples were randomly collected from major cities (Chennai, Coimbatore, Madurai, Trichy, and Salem) of Tamil Nadu state, India and analyzed for Pb, Cd, As and Hg residues (mg/kg) using inductively coupled plasma-mass spectrometry (ICP-MS) technique. The results revealed that lead (Pb) concentration was higher in meat and liver samples of all cities and the highest concentration was detected in Chennai followed by Coimbatore, whereas in kidney and neck samples, Chennai metropolitan alone exceeded the MRL value of 0.5 and 0.1 mg/kg respectively. The residues of cadmium and mercury in meat and meat samples were within the permissible limit in all the metropolitans. Arsenic was found exceeding the MRL of 0.1 mg/kg in the breast samples of all the metropolitans (0.2141–2.2285 mg/kg, the highest being in Trichy), except for Madurai, which recorded very low value (0.0239 mg/kg). Arsenic was found in the range of 0.1110–1.0850 mg/kg in liver samples thus exceeding MRL value. Neck and kidney samples did not contain any Arsenic. Thus it can be concluded that the lead concentration in meat and edible organ samples collected from all the metropolitans exceeded the MRL values and is an indication of contamination. Appropriate precautions are warranted to minimize the heavy metal consumption contamination especially of Pb and As.

## Highlights

• Pb exceeded maximum residue limit in meat and liver in all 5 cities of Tamil Nadu state of India while kidney and neck samples of Chennai alone exceeded maximum residue limit.
• As was found to be exceeding maximum residue limit in breast (0.1 mg/kg) and liver (0.5 mg/kg) samples in many metropolitans of Tamil Nadu state.
• Cd and Hg concentration was within the maximum residue limit in all metropolitan samples.
• Daily dietary consumption of Pb, Cd, As and Hg from edible parts of chicken in Tamil Nadu metropolitans was well within the permitted quantity.

## Graphical abstract

Possible contaminant route of heavy metals in human through chicken consumption.

**Figure:**
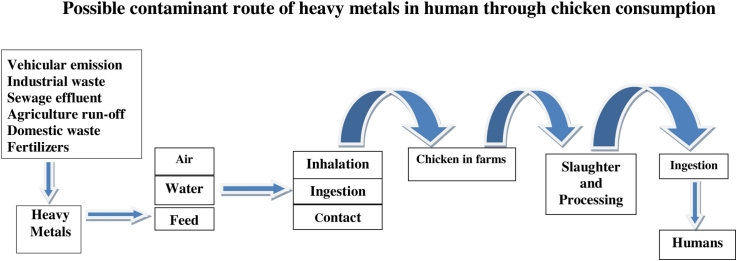

## 1 Introduction

Chicken meat production in India has been phenomenally increasing with the production of 4.855 million tons of ready to cook equivalent registering a growth of 17.98 % in 2018 from 2015 [1]. Chicken meat is comparatively cheaper than other meats and occupies about 45 % of total meat consumed in India [2]. The current human population of 1.36 billion is poised to reach 1.50 billion in 2030 and 75 % population of 15 years old and above is found to be meat-eating [3] and much of the meat requirement can only be met with chicken. Faster industrialization with effluent releases, intensified agriculture activities using inorganic fertilizers, larger vehicular emissions and dumping of municipal solid wastes and contamination of water bodies are most likely to contaminate the food chain including the intensified chicken meat production in India. Globally, food contamination with heavy metals is considered a serious health concern and particularly in cities of major developing countries like India [4]. These heavy metals are ubiquitous and chemically stable [5], non-biodegradable, have a longer biological half-life and accumulate in the tissues causing stern threats to the food chain [6]. Lead and cadmium contamination in food items like fish in River Ganga Basin, India, has been reported exceeding safe limits [7]. River waters have been reported to contain appreciable concentrations of cadmium, arsenic, lead and mercury in Ghana [8] and cadmium and lead in India [7]. A few or more heavy metals contamination was increasingly reported in various food items like over-the-counter food supplements like protein powder [9], vegetable oils [10] and walnut [11] that has become a cause of concern in human nutrition.

Ingestion of toxic metals even at lower concentrations over a long period leads to severe health issues. These elements accumulate in different body parts and mainly affect the reproduction and growth [12] and often have direct physiological toxic effects [13]. Apart from affecting various biological systems like central and peripheral nervous, digestive and genital systems, toxic metals have been reported to even take part in antimicrobial resistance formation in human beings indirectly by affecting the immune system [14,15]. High lead concentration in food has been reported to be associated with cardiovascular, renal, nervous, and skeletal-system diseases [16,17]. Chronic lead exposure causes developmental abnormalities, deficits in intelligence quotient, neurotoxicity in infants, constipation, colic and anaemia [18].

Food is the primary source of cadmium exposure and its adverse health effects are kidney and bone damage. Being absent at birth, cadmium starts accumulating in the human body and causes damage to all body systems [19]. Arsenic may reach meat through drugs used in poultry production [20]. Though acute arsenic toxicity is very rare, it is frequently characterized by severe gastrointestinal, cardiovascular, central nervous system related ailments and death [21]. Moreover, consumption of tissues with inorganic arsenic residues increases the incidence of bladder and lung cancer in human beings [22]. Mercury can cause damage to the renal tubules and on continuous exposure, elemental Hg can accumulate in the thyroid [23]. Carcinogenic and noncarcinogenic adverse effects are caused by nonessential trace metals such as arsenic, cadmium and lead even at low concentrations [24,25].

The objective of the present study is to ascertain the concentration of heavy and toxic metals like lead (Pb), cadmium (Cd), arsenic (As) and mercury (Hg) in common edible broiler chicken parts (breast, liver, neck, and kidney) collected from the major metropolitan cities of Tamil Nadu, India, in order to primarily assess the status of contamination of these toxic metals, in the light of the fact that chicken continues to dominate the non-vegetarian food of Indians.

## 2 Materials and methods

### 2.1 Site selection and sample collections

**Fig. 1:**
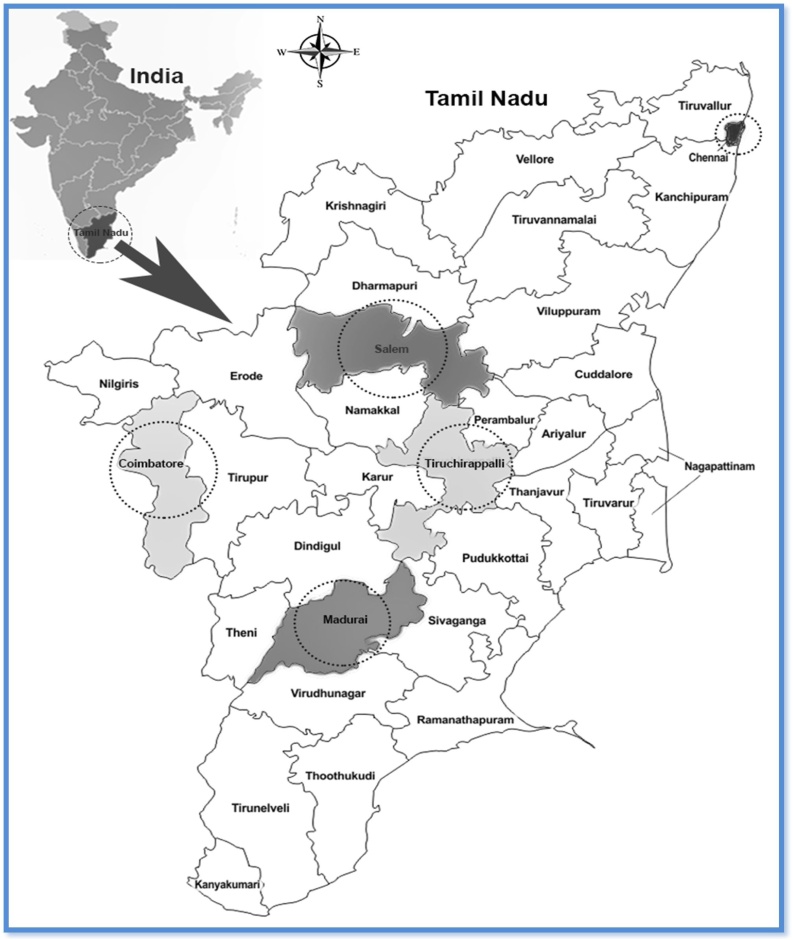
Map indicating the metropolitan cities where live chicken were samples in Tamil Nadu, India.

**Fig. 2:**
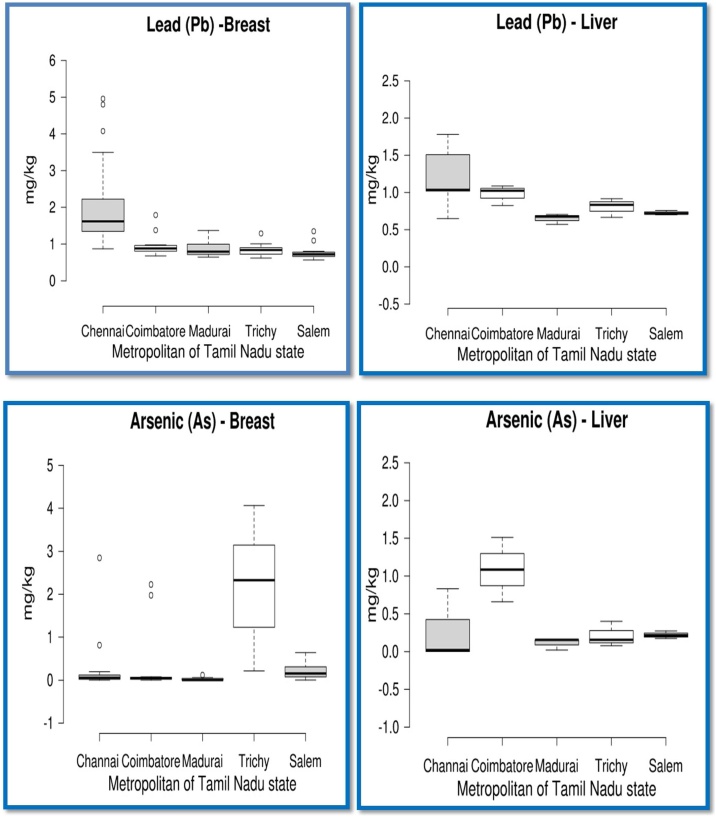
Concentration of Pb and As (mg/kg) in broiler meat and liver samples collected from metropolitans of Tamil Nadu state, India. Lower line represents first quartile, middle line represents second quartile and upper line indicates third quartile. Upper and lower whiskers indicate maximum and minimum values which excluded outliers. For neck and kidney, the values were narrower for Pb, while As was not detected.

The sites for sampling of chicken in the state of Tamil Nadu were selected in places where the chicken consumption is high [1]. An assessment was made to estimate four major toxic (Pb, Cd, As and Hg) metals in chicken breast, liver, kidney, and neck portions. The samples of chicken parts were collected from live chicken slaughter shops in five highly populated metropolitan cities of Tamil Nadu (Chennai, 8.69 million; Coimbatore, 2.15 million; Madurai, 1.46 million Tirchy, 1.02 million and Salem, 0.96 million, [50]; Figs. 1 and 2 ) and shops were chosen randomly in different sectors of the cities. Samples were collected between January and September 2017. Ceramic knives were exclusively used for cutting the chicken parts to avoid metal contamination. Samples were immediately packed, labeled, and stored in a cooler box with an ice pack and transferred to a locally arranged freezer in each city and transferred to the place of analysis and stored in a freezer at −20 °C until further analysis (Table 1).

**Table 1:** Collection of chicken samples (January to September-2017).

S. No	Chicken parts	Chennai	Coimbatore	Madurai	Trichy	Salem	Total
1	Meat	30	12	12	12	12	78
2.	Liver	10	3	3	3	3	22
3.	Kidney	5	5	5	5	5	25
4.	Neck	5	5	5	5	5	25
Total		50	25	25	25	25	150

### 2.2 Standards, reagents and apparatus

The NIST traceable standards (catalog no. Pb, N9304320; Cd, N9300107; As, N9300102; and Hg, N9304326) were purchased from *Perkin Elmer* (USA) with the concentration of 1000 mg/L. MS grade nitric acid 65 % and hydrogen peroxide 30 % procured from Merck, India, were used for digestion of samples. Standard class A measuring flasks (25 mL, *Borosil*, India) and Handystep multivolume pipette (*Brand*, India) were used in the study. Samples were weighed in calibrated electronic balance (*Mettler Toledo*, India) with 0.1 mg accuracy. Digestion was carried out using *CEM* Corporation *MARS 5* digestion microwave system. Estimation was done using Inductively Coupled Plasma-Mass Spectrometer (ICP-MS 7700, *Agilent,* USA) at The Micro Therapeutic Research Labs Private Limited, Chennai, accredited by FDA.

### 2.3 Standard calibration curve preparation

Appropriate metal standards of Pb, Cd, As and Hg were diluted with 1% nitric acid to an initial concentration of 10 μg/mL. These initial standards were further diluted with 2% nitric acid to arrive at 500 ng/mL of each element, which was the intermediate working standard. They were further sub-diluted to different smaller concentrations for calibration in ICP-MS instrument as per the standard concentration and instrument parameters and are shown in Table 2, Table 3.

**Table 2:** Concentrations used for calibration curve in ICP-MS for the heavy metals analysis.

Toxic Metals	Standard concentrations ng/mL							
Lead	0.00	2.5	5	10	20	40	80	100
Cadmium	0.00	2.5	5	10	20	30	40	50
Arsenic	0.00	2.5	5	10	20	40	80	100
Mercury	0.00	2.5	5	10	20	30	40	50

**Table 3:** ICP-MS parameters for heavy metal analysis in chicken meat.

Parameters	Time (Sec)	Speed (rotation per second) of Nebulizer pump
Sample uptake	20	0.3
Stabilize	40	Tune parameter
**Tune parameters**		
Probe rinse (sample)	20	0.3
Probe rinse (std)	10	
Rinse 1	10	0.3
Peak Pattern	3 Points	
Replicate	1	
Tune mode	Helium gas (He)	
Plasma mode	Low Matrix	

### 2.4 Sample digestion and chemical analysis

Before sample digestion, the glassware were washed with triple distilled water. They were then soaked overnight in 5% HNO3 solution overnight for 12 h and rinsed in Millipore (18.2 micro-ohms) water a few times before the actual use. The chicken samples were thawed under room temperature and cut into small pieces using a ceramic knife. About 50 g of the sample was transferred into pestle and mortar and thoroughly homogenized. 0.500 g of sample was weighed precisely and transferred into microwave digestion vessels (XP-1500 Plus). Thereafter, 7 mL of 65 % high pure nitric acid was added and allowed to wait for 10 min until the yellow fumes subsided. Further, 3 mL of 30 % hydrogen peroxide was added [26]. The vessels were sealed and placed into the microwave digestion system. The samples were mineralized following a set pattern of temperature program (Table 4). A clear solution of the digested sample was obtained and quantitatively transferred into 25 mL volumetric flasks, labeled and analyzed for its mineral contents. A blank was maintained and treated the same way as for the samples during the digestion process.

**Table 4:** Sample digestion programme in the digester (Mars 5).

Step	Temperature	Pressure max (Psi)	Ramp time	Hold time
1	210	350	10	10
2	50	40	1	0

### 2.5 Quality assurance

Instrument performance was checked with ICP-MS tune solution (Agilent part no: 5184 - 3566) and was found satisfactory. Validation and sample analyses were performed as per Food and Drug Analysis guidelines [49]. Replicate analysis of the reference materials showed acceptable precision (RSD, Pb<7.0; Cd <1.49; As <2.44 and Hg< 1.39), recovery (Pb, 89.60; Cd, 92.51; As, 94.52 and Hg, 90.21 %) and repeatability (Pb, 99.93; Cd, 97.78; As, 97.45 and, Hg 97.78 %). Seven point calibration graphs were set up at the linear range of 2.5–100 ppb for Pb and As and at 2.5−50 ppb for Cd and Hg (Table 2) with acceptable correlation coefficient (0.9998, 1.000, 0.9997 and 0.9975 for Pb, Cd, As and Hg respectively). The limit of detection and limit of quantification were 0.18, 0.95, 0.21 and 0.25 and 1.85, 1.95, 2.10 and 2.5 ppb for Pb, Cd, As and Hg respectively.

### 2.6 Statistical analysis

The statistical analysis was performed with SPSS software version 20. Mean concentration of values obtained from this study was done by one way analysis of variance (ANOVA). Differences between means were compared by Tukey’s method and considered significant at 5% (p < 0.05). The results are presented as means with standard error of mean and p value. Superscripts have been placed wherever means were significant at 5% and 1% [27].

## 3 Results and discussion

### 3.1 Lead (Pb)

**Fig. 3:**
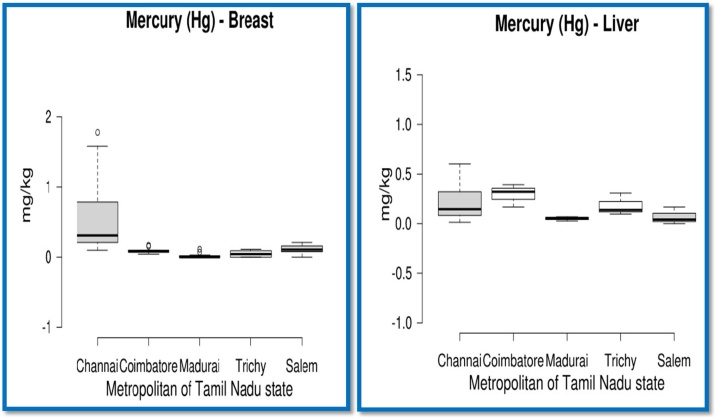
Concentration of Hg (mg/kg) in broiler meat and liver samples collected from metropolitans of Tamil Nadu state, India. Lower line represents first quartile, middle line represents second quartile and upper line indicates third quartile. Upper and lower whiskers indicate maximum and minimum values which excluded outliers.

The mean concentration of Pb (mg/kg) found in the edible parts such as breast, liver, neck and kidney of the chicken samples is presented in Table 5 and depicted in Fig. 3. The concentration of Pb detected in the breast samples collected from Chennai, Coimbatore, Madurai, Trichy and Salem metropolitan cities was found to be 1.9626, 0.9700, 0.8887, 0.8428, and 0.7808 mg/kg, respectively. Pb concentration in breast from Chennai city was significantly higher (p < 0.05) than in breast samples from other cities and the concentration for breast samples was in the order of Salem < Trichy < Madurai < Coimbatore < Chennai. Though, rest of the chicken edible parts did not show any significant difference amongst the cities, Chennai samples continued to show highest concentration of Pb (mg/kg) in liver, neck and kidneys than in samples obtained from other cities. All the breast samples collected were found to contain Pb irrespective of metropolitans and clearly exceeded the MRL value of 0.1000 mg/kg concentration stipulated by many regulatory bodies (Table 6) and similar trend was observed in liver samples also across all the cities exceeding the 0.5000 mg/kg MRL value. Similarly, samples of neck and kidney from Chennai showed higher values than the stipulated MRL value but other cities did not.

**Table 5:** Pb, Cd, As and Hg concentrations (mg/kg) in edible. chicken parts in major metropolitans of Tamil Nadu.

Heavy metals	Chennai	Coimbatore	Madurai	Trichy	Salem	P value	Min ∼ Max	Overall Mean
**Lead (Pb)**								
Breast	1.9626b ± 0.198 (n = 30)	0.9700a ±0.093 (n = 12)	0.8887a ± 0.067 (n = 12)	0.8428a ± 0.053 (n = 12)	0.7808a ± 0.064 (n = 12)	0.001	0.5633- 4.9577	1.2906b (n = 78)
Liver	1.1689 ± 0.108 (n = 10)	0.9788 ± 0.078 (n = 3)	0.8086 ± 0.040 (n = 3)	0.8050 ± 0.073 (n = 3)	0.7580 ± 0.017 (n = 3)	0.079	0.6475−1.7798	0.9882b (n = 22)
Neck	0.2604 ± 0.252 (n = 5)	0.0115 ± 0.002 (n = 5)	0.0801 ± 0.046 (n = 5)	0.0119 ± 0.001 (n = 5)	0.0245 ± 0.006 (n = 5)	0.508	0.0048−1.2690	0.0777a (n = 25)
Kidney	0.8582 ± 0.824 (n = 5)	0.0716 ± 0.054 (n = 5)	0.0070 ± 0.001 (n = 5)	0.0059 ± 0.001 (n = 5)	0.0129 ± 0.002 (n = 5)	0.419	0.0017- 4.1546	0.1911a (n = 25)
**Cadmium (Cd)**								
Breast	0.0194ab ± 0.00 (n = 30)	0.0177a ± 0.00 (n = 12)	0.0185ab ± 0.00. (n = 12)	0.0191ab ± 0.00 (n = 12)	0.0204b ± 0.00 (n = 12)	0.021	0.0142 - 0.0261	0.0191 (n = 78)
Liver	0.0176a ± 0.00 (n = 10)	0.0186ab ± 0.00 (n = 3)	0.0214b ± 0.000 (n = 3)	0.0187ab ± 0.00 (n = 3)	0.0200ab ± 0.00 (n = 3)	0.010	0.0133−0.0222	0.0187 (n = 22)
Neck	0.0584 ± 0.036 (n = 5)	0.0003 ± 0.000 (n = 5)	0.0005 ± 0.000 (n = 5)	0.0005 ± 0.000 (n = 5)	0.0086 ± 0.005 (n = 5)	0.087	0.0000−0.1642	0.0137(n = 25)
Kidney	0.0707 ± 0.068 (n = 5)	0.0059 ± 0.004 (n = 5)	0.0009 ± 0.000 (n = 5)	0.0008 ± 0.000 (n = 5)	0.0010 ± 0.00 (n = 5)	0.424	0.0004−0.3430	0.0159 (n = 25)
**Arsenic (As)**								
Breast	0.3897a ±0.134 (n = 30)	0.3833a ± 0.232 (n = 12)	0.0239a ± 0.010 (n = 12)	2.2285b ± 0.387 (n = 12)	0.2141a ± 0.058 (n = 12)	0.001	0.0014 - 4.0655	0.5883 (n = 78)
Liver	0.2025 ± 0.100 (n = 10)	1.0850 ± 0.245 (n = 3)	0.1110 ± 0.045 (n = 3)	0.2806 ± 0.015 (n = 3)	0.2208 ± 0.028 (n = 3)	0.003	0.0014−1.5111	0.7781 (n = 22)
Neck & Kidney	ND	ND	ND	ND	ND	–	–	–
**Mercury (Hg)**								
Breast	0.5457b ± 0.089 (n = 30)	0.0908a ± .0109 (n = 12)	0.1454a ± 0.091 (n = 12)	0.1082a ± 0.180 (n = 12)	0.0477a ± 0.012 (n = 12)	0.001	0.0000 - 1.7782	0.2702 (n = 78)
Liver	0.2109 ± 0.055 (n = 10)	0.2941 ± 0.066 (n = 3)	0.0513 ± 0.0122 (n = 3)	0.1815 ± 0.064 (n = 3)	0.0701 ± 0.050 (n = 3)	0.202	0.0000 - 0.6015	0.1773 (n = 22)
Neck & Kidney	ND	ND	ND	ND	ND	–	–	–

n−number of observations; ND−not detected; Mean values bearing different superscripts in a row differ significantly (p<0.05).

**Table 6:** Maximum permissible levels (mg/kg) limits in chicken meat by worldwide regulatory bodies.

Name of the Regulatory body		Country	Lead		Cadmium		Arsenic		Mercury
[40]	Australia New Zealand Food Authority, 2015	Australia & New Zealand	Meat-0.1 Offal-0.5	–		–		–	
[41]	Codex Alimentarius, 2009	United States	Meat-0.1	–		–		–	
			Offal-0.5						
[42]	Export Inspection Council India, 2017	India	Meat-0.1 Offal-0.5	Meat-0.05 Liver-0.5 Kidney-1.0		–		–	
[43]	European Communities, 2006	European Countries	Meat-0.1	Meat-0.05 Liver-0.5		–		1.0	
[44]	Food Safety and Standards	India	Meat-0.1	–		–		–	
	Authority of India, 2011		Offal-0.5						
[45]	Food Safety Authority of Ireland, 2009	Ireland	Meat-0.1 Offal-0.5	Meat-0.05 Liver -0.5 Kidney-1.0		–		–	
[46]	FAO/WHO, 2002	–	0.1	Meat-0.05		–		–	
				Liver-0.5					
[47]	CN, 2005	China	0.2	Meat-0.1 Liver-0.5		–		–	
[48]	JECFA, 2005	–	0.1	0.1		0.1		–	

Highest individual value of 4.9577 mg/kg in a breast sample and maximum average value of 1.9626 mg/kg of Pb were found in breast samples of Chennai followed by next high Pb residual values in other cities. This indicated a high level of Pb contamination and theses values were clearly far higher than values reported in meat samples in various countries [5,28,29]. A report on Pb concentration in chicken collected from different markets in Kolkata city in India showed very high value of 7.11 mg/kg [4]. The authors attributed the high Pb level to location of market under a busy traffic flyover and selling of food items in the open. High Pb concentration in atmospheric air of residential (40 ng/m3) and industrial (118 ng/m3) areas of Kolkata in India [30], could be a source of Pb contamination. Such high Pb concentration is quite possible in every metropolitan of India. Pb concentration exceeding the MRL value in chicken meat [31] and offal [32] was reported earlier in Bangladesh and Iran respectively. However, a detailed trend analysis of monitoring results carried out in Netherlands [33] showed that the possible Pb contamination of feeds could originate from using feed ingredients of mineral origin and toxin binders of clay origin. In the present study, chicken samples from Chennai city showing higher Pb contamination could be mostly due to vehicular emission and refining industries that located in Chennai whose number is more than in other cities.

In an attempt to characterize the sources of PM10 and PM2.5 in Chennai city [51], high concentration of Pb in PM10 and PM2.5 was reported to be from industrial source as well as from marine aerosol source (in PM2.5) as Chennai is situated on the east coast of Bay of Bengal. The evidence that Pb contamination in agricultural soils to the tune of 55–77 % from atmospheric deposition in the soils of England also strongly suggests the possibility of higher Pb residue deposition in chicken samples of all the metropolitans studied [14]. Mostly, the slaughter of chicken was done in open fashioned stalls situated in busy vehicular traffic areas. Recent global survey [34] shows that 15 % of poultry feeds in excess of EU limit (5 ppm) for Pb, 17 % of individual mineral samples in excess of EU limit (100−400 ppm) and 4 % of mineral premixes in excess of EU limit (< 200 ppm) also suggests another possible route of Pb contamination in chicken.

### 3.2 Cadmium (Cd)

Data pertaining to the observed mean concentration of Cd in breast, liver, neck and kidney samples are presented in Table 5. Breast samples in Chennai, Coimbatore, Madurai, Trichy, and Salem showed Cd concentrations (mg/kg) of 0.0194, 0.0177, 0.0185, 0.0191, and 0.0204 respectively. Though breast samples from Salem showed significantly (p < 0.05) higher Cd contamination, it was significantly higher only by a margin over the value recorded in breast samples of Coimbatore city. However, the concentrations were well within the MRL values of 0.05 mg/kg in all the collected breast samples as prescribed by the regulatory body of India (Table 6). Cd concentrations (mg/kg) in neck samples were found to be 0.0584 (Chennai), 0.0003 (Coimbatore), 0.0005 (Madurai), 0.0005 (Trichy) and 0.0086 (Salem), which were all well within the MRL values. The samples of liver and kidney also showed very low Cd contamination in all the 5 metropolitans of Tamil Nadu, suggesting of low level of Cd contamination in all the possible routes like feeds and atmosphere, as additionally supported by the recording of absence of Cd in particulate matters in Chennai [51].

It could be observed that the Cd contamination was generally low amongst all the samples in all the five metropolitans and hence may be considered safe for consumption. Cd contamination was reported to be high even in edible parts of (free range chicken) in areas where mining activities were plenty [35,36] but was within permissible quantity in chicken giblets collected from retail markets of Egypt [37] and in meat collected from the biggest metropolitan city of Dhaka, in Bangladesh [38]. Moreover, it was reported that chicken meat showed high Cd content (1.36–1.68 mg/kg) in Saudi Arabian city, Riyadh [39].

### 3.3 Arsenic (As)

The results of As concentration (mg/kg) measured in different edible parts of chicken are presented in Table 5 and depicted in Fig. 3. The concentration of As was found to be 0.3897, 0.3833, 0.0239, 2. 2285 and 0.2141 mg/kg in breast samples collected from Chennai, Coimbatore, Madurai, Trichy and Salem cities, respectively. Comparison between cities revealed that significantly high As concentration (p < 0.05) was observed in Trichy city while samples from other cities recorded low but exceeding MRL (Table 6) values. As concentration in liver samples was high (1.0850 mg/kg) in samples collected from Coimbatore city while in the samples collected from the rest of the cities recorded values between 0.1110 and 0.2806 mg/kg. As was not detected in the neck and kidney samples collected from all five metropolitans. Generally, As concentration exceeded the MRL value of 0.1 mg/kg (Table 6) in all the breast and liver samples of metropolitans except for Madurai (breast, 0.0239 mg/kg). Chicken samples were reported to have lower As concentration in the areas near the mines (0.04 mg/kg, [36]) or in areas near the vicinity to industries in Bangladesh (0.032 mg/kg [38] and in various chicken products in Turkey [26]. However, in India, chicken samples were shown to contain 0.14 mg/kg in urban Kolkata [4]. Though the reason for high As concentration in breast and liver samples of chicken collected from majority of cities was not known, probably As contamination could have made entry by atmospheric deposition [14] or possibly from the poultry feeds as globally assessed [34] to be exceeding EU limit of 5 ppm in 19 % of poultry feeds in an elaborate study on feeds for heavy metals contamination, worldwide.

### 3.4 Mercury (Hg)

Mean Hg concentration (mg/kg) in breast and liver samples is presented in Table 5 and depicted in Fig. 3. The analyzed values in Chennai, Coimbatore, Madurai, Trichy and Salem metropolitans were 0.5457, 0.0908, 0.1454, 0.1082 and 0.0477 mg/kg, respectively. The results revealed a significantly (p < 0.05) higher concentration in Chennai city (maximum of 1.7782 mg/kg in one sample). The order of Hg concentration in breast samples was Salem < Coimbatore < Trichy < Madurai < Chennai. The mean Hg concentration present in liver samples was found to be 0.2109 (Chennai), 0.2941 (Coimbatore), 0.0513 (Madurai), 0.1815 (Trichy) and 0.0701 (Salem). The concentration of Hg in liver samples did not differ significantly. The Hg concentrations in neck and kidney samples were recorded to be below the detectable levels. Though the average Hg content detected in breast samples collected from Chennai was below the MRL value of 1.0 mg/kg (Table 6), a few samples recorded values exceeding the MRL. Chicken organs, but not chicken meat, showed Hg concentration to the level of 0.11−0.12 mg/kg in free range chicken in Ghana [36] but not in chicken samples in certain districts of Saudi Arabia (0.009−0.015 μg/g) dry weight, [39].

### 3.5 Toxicological risk

Daily intake (mg/day/person) of all the four toxic metals through consumption of chicken breast in each metropolitan of Tamil Nadu state is given in Table 7 and compared against maximum permitted daily dietary allowance per person [52]. Chicken breast alone was taken into account for calculating the daily intake of toxic metals, as the consumption of breast is highest amongst the meat and organs sampled in this study. The results showed that none of the metals exceeded the maximum daily dietary allowance permitted, although individual toxic metals like Pb and As exceeded the MRL value. This signified that there is no toxicological risk associated with the intake of any metals taken in this study.

**Table 7:** Comparison of daily intake of toxic metals from chicken breast in metropolitans of Tamil Nadu with the recommended daily dietary allowance per person [52].

Toxic metal		Pb	Cd	As	Hg
Recommended daily dietary allowance (mg/day /person)		0.21	0.06	0.13	0.03
**Metropolitans**	Monthly urban per capita consumption of chicken in Tamil Nadu [53]	Calculated value daily dietary intake from chicken (mg/day/person)			
**Chennai**	0.278 kg	0.0182	0.0002	0.0036	0.0051
**Coimbatore**		0.0090	0.0002	0.0036	0.0008
**Madurai**		0.0082	0.0002	0.0002	0.0013
**Trichy**		0.0078	0.0002	0.0207	0.0010
**Salem**		0.0072	0.0002	0.0020	0.0004

## 4 Conclusions

The extent and distribution of toxic heavy metals Pb, Cd, As and Hg in broiler meat and chicken parts neck, liver and kidney were estimated in samples collected from five major metropolitans of state of Tamil Nadu, India. It was found that the concentration of Pb exceeded the MRL values in breast (0.1 mg/kg) and liver (0.5 mg/kg) samples collected from all the metropolitan cities. The order of Pb concentration found in breast and liver samples was Salem < Trichy < Madurai < Coimbatore < Chennai. Cd contamination in all samples of all the metropolitans was lower than MRL values of 0.05 mg/kg. As contamination in breast and liver samples collected from majority metropolitans exceeded the limited value of 0.1 mg/kg. The order of concentration of Arsenic in breast meat of broilers was Madurai < Salem < Coimbatore < Chennai < Trichy. However, the concentration of mercury was found to be within the MRL of 1.0 mg/kg in the breast and liver samples collected from metropolitans of Tamil Nadu. Hence forth, it was observed that chicken meat and organs sampled in 5 metropolitans of Tamil Nadu state of India contained concentration exceeding MRL values for Pb and As (except for Madurai for As) but low concentrations of Cd and Hg in all the metropolitans. This study suggests a widespread analysis of atmospheric air and poultry feeds in order to identify the source to avoid the further contamination. Routine analysis of feed materials and feeds should form a basis of quality control for detecting heavy metals in edible parts of chicken in India.

## Declaration of Competing Interest

The authors declare that they have no known competing financial interests or personal relationships that could have appeared to influence the work reported in this paper.
